# Supplementary material for: Is household contact investigation a missing link for tuberculosis care in Chhattisgarh, India?–Operational research using programmatic data
Source: PLOS Glob Public Health. 2025 Sep 24;5(9):e0005246. doi: 10.1371/journal.pgph.0005246 (PMC12459760; doi:10.1371/journal.pgph.0005246)
Supplement: Attachment — Submitted filename: Response letter to reviewers comments.docx [file pgph.0005246.s003.docx]

PGPH-D-25-00886	June 2025
Is household contact investigation a missing link for tuberculosis care in Chhattisgarh, India? – Operational research using programmatic data
PLOS Global Public Health

**Johanna Pruller, Ph.D.
PLOS Staff Editor
PLOS Global Public Health**

Subject- Point-point response to the reviewer’s comments

Thank you for the email communication on 30^th^ May 2025 and providing comments and suggestions from the reviewer. We have carefully read all the comments and made relevant changes in the manuscript. We have attempted to respond to each of the points raised. The reviewer’s comments are copied below, and the responses provided point by point.

We also used this opportunity to go through the manuscript again and make editorial changes to improve readability.

We hope that the responses in the letter and changes made in the revised manuscript are to the satisfaction of the reviewer and editor. We appreciate the effort put in by both and providing us a valuable peer review to improve the manuscript.

Yours sincerely,

Chetanya Malik

Corresponding author

COMMENT

In the results, “of the 4221 households having an adult with bacteriologically confirmed pulmonary TB (index) between October to December 2023, contact investigation was conducted for 75% (n=3177). Aggregate contact tracing register data showed a total of 11670 contacts being screened and of them, TB was diagnosed in 0.9% (n=109) and TPT initiated for 66% (n=7740) contacts”.

The study aims to assess the coverage of HHC investigation. However, for the scope of the study, it could only report the proportion of households having bacteriologically confirmed pulmonary TB have contact investigation done (75), not report the proportion of number of HHC (11670 contacts) being screened among total number of HHC (of 4221 PTB index cases); and also the proportion of number of HHC (11670) being screened among total number of HHC of 3177 index cases (we could not sure that 100% of HHC of 3177 index cases being screened). This is need to be discussed in the discussion and mention in the limitation if could not provide this information.

RESPONSE

For this study we operationally defined HHC investigation not done as "After merging the databases, if there were no numbers (aggregate) reported in the contact tracing register, it was recorded as not undergoing household contact investigation". This is mentioned in lines 146-148 of the revised manuscript with track changes. Thus, the total HHCs documented for 3177 index cases was 11670. Please note this is the documented HHCs and may or may not be total HHCs for 3177 index cases. For the remaining 1044 index cases, whose data was not entered, we mentioned it as HHC investigation not done as per the operational definition and thus do not know the HHCs of these index cases. Against each notified person in the Ni-kshay notification register, aggregate numbers of HHC are documented in the contact tracing register by the TB program staff. These may or may not be the total number of HHCs. Hence. we could not assess the proportion of HHC that were screened. These points are discussed in lines 177-178 and 229-232 of revised manuscript with track changes.

COMMENT

The study result shows TB was diagnosed in 0.9% (n=109) among HHC being screened. This is high proportion, meaning prevalence of 934 per 100,000 (compare to 129 per 100,000 PTB notification; 373 per 100,000 TB prevalence in this region). However, line 49, “low yield of TB”, please clarify.

RESPONSE

The “low yield of TB” has been inferred from studies mentioned in lines 239-245 of the revised manuscript with track changes, which includes a meta-analysis. These studies have documented 3-5% of HHCs being diagnosed with TB disease.

COMMENT

The study aims to assess the coverage of HHC investigation, proportions identified with TB and put on TPT for all age groups and age<5 years. As the results, of 11670 contacts screened, TB was diagnosed in 0.9% (n=109) and TPT initiated for 66% (n=7740) contacts. Of the identified contacts, 1230 were below the age of 5 years; of whom nine (0.7%) were diagnosed with TB and 73% (n=903) were initiated on TPT. May more detail analysis and present in another table for the comparison of proportion of identified TB in different groups.

RESPONSE

As shared in the manuscript, the denominator was those with pulmonary TB notified in the *Ni-kshay* notification register who were tracked in the contact tracing register (electronic module in Ni-kshay) as to whether aggregate numbers of contacts were identified, had undergone TPT and diagnosed with TB. Hence, other than analysis of index level characteristics, we were not able to do further analysis. Among those notified with pulmonary TB, what proportion did not undergo HHC investigation and what are the risk factors of not undergoing HHC investigation has been analysed. We wanted to do a detailed analysis of comparison of proportion of TB detected among different subgroups of contacts. As we do not have individual level data of contacts identified (only aggregate number available against an index TB in contact tracing register), we are not able to do a detailed analysis of which subgroup of contacts had a higher proportion of TB detection (in adults and in child contacts). These have been mentioned in lines 152-153 and lines 279-285 of revised manuscript with track changes. We hope this is fine.

COMMENT

Table 1, the percentage are row percent, but the clinical characteristic group the percentage are column percent (i.e. new (71.6%); resistant (7.5%), please check and correct.
RESPONSE

Thank you very much for pointing this out. In first column it is column percentage and then it is row percentage. We have corrected the typing errors in table 1 using track changes

COMMENT
Of 78 HHC of resistant cases, all 78 (100%) was not being screened for TB. This should be highlight and discuss for improvement. (This proportion was present as column percent in table 2, should be row percent as mentioned above).

RESPONSE

Thank you for the comment and suggestions. We have discussed this in lines 265-271 in the revised manuscript with track changes. The reason for this is- “diagnosis and treatment of those with drug resistant TB happens in tertiary care settings and TPT for drug resistant TB has been recently introduced”. We have corrected the column and row percentages in the tables (apologies for the typing error).
