# Supplementary material for: Is household contact investigation a missing link for tuberculosis care in Chhattisgarh, India?–Operational research using programmatic data
Source: PLOS Glob Public Health. 2025 Sep 24;5(9):e0005246. doi: 10.1371/journal.pgph.0005246 (PMC12459760; doi:10.1371/journal.pgph.0005246)
Supplement: Attachment — Submitted filename: Response letter to reviewers comments_v3.docx [file pgph.0005246.s004.docx]

**PGPH-D-25-00886	July 2025**

Is household contact investigation a missing link for tuberculosis care in Chhattisgarh, India? – Operational research using programmatic data

PLOS Global Public Health

**Sonali Sarkar
Academic Editor
PLOS Global Public Health**

**Subject**- Point-point response to the reviewer’s comments

Thank you for the email communication on 17^th^ July 2025 and providing comments and suggestions from the reviewer. We have carefully read all the comments and made relevant changes in the manuscript. We have attempted to respond to each of the points raised. The reviewer’s comments are copied below, and the responses provided point by point.

We also used this opportunity to go through the manuscript again and make editorial changes to improve readability.

We hope that the responses in the letter and changes made in the revised manuscript are to the satisfaction of the reviewer and editor. We appreciate the effort put in by both and providing us a valuable peer review to improve the manuscript.

Yours sincerely,

Chetanya Malik

Corresponding author

COMMENT

The outcome variable - of HHC investigation not done is a dichotomous variable. Why is Poisson regression model used instead of logistic model.

RESPONSE

We have used modified Poisson regression model as mentioned in line 156 for this study (attached is the reference article for using this model in case of Binary outcomes).

Considering the design of the study (cross-sectional analytical study), prevalence ratio was the most appropriate measure of association. Logistic regression provides odds ratio (not prevalence ratio). Additionally, when the outcome is not rate, odds ratio overestimates the risk and prevalence ratio is preferred. Hence, prevalence ratio is the preferred choice of measure of association. It is also easier to interpret for the people in the program.

We tried generating prevalence ratio using log binomial regression but the model did not converge. Hence, we then used modified poisson regression to estimate the prevalence ratio after adjusting for confounders.

We hope this clarifies.

I hope this is fine.

COMMENT

Line no. 40-41, please indicate number and percentage

RESPONSE

The numbers and percentage are mentioned in the lines 40-42 of the revised manuscript. The lines now read as “Among total11670 contacts screened, TB was diagnosed in 0.9%(n=109) for all age groups and 0.7%(n=9) for children<5 years. TPT was initiated in 66% (n=7740) for all age groups and 73% (n=903) for children<5 years.”

COMMENT
 Line no. 172, Is contact tracing a better word?

RESPONSE

Contact investigation has been the term used in World Health Organization and National TB Program documents of India and has thus been used in the manuscript.

COMMENT
 Lines no. 228 - 233 - Unclear what authors are trying to convey. Please rephrase

RESPONSE

The lines 228-233 have been rephrased in lines 228 to 234 to convey the limitation of lack of availability of data pertaining to all household contacts. The lines now read as “ This being operational research using available secondary data from the state’s TB program, the aggregate number of household contacts entered in the database were used for the study. This may not be all the contacts of those notified with TB but only aggregate numbers of HHCs entered in the online portal. Secondly as HHC investigation was done for 75% (3177 out of 4221) of those notified as per the portal, the aggregate number of reported HHCs are those for whom data is entered. Thus, we could not know the proportion of HHC screened out of total contacts but only those documented as aggregate numbers.” Only the data entered in the portal for HHCs is available and hence what proportion of household contacts were screened could not be calculated.

COMMENT
 Line no. 240 - Please mention to which geographic context does this meta-analysis refer to?

RESPONSE

The geographical context has been mentioned in lines 242-243.

COMMENT
 Line no. 262-263 - How is this point substantiating lack of follow-up among patients diagnosed in secondary / tertiary facilities?

RESPONSE

This has been rephrased in lines 259-261 of the revised manuscript to substantiate lack of follow-up among patients diagnosed in secondary/tertiary facilities.
