# Supplementary material for: Is household contact investigation a missing link for tuberculosis care in Chhattisgarh, India?–Operational research using programmatic data
Source: PLOS Glob Public Health. 2025 Sep 24;5(9):e0005246. doi: 10.1371/journal.pgph.0005246 (PMC12459760; doi:10.1371/journal.pgph.0005246)
Supplement: Attachment — Submitted filename: Response letter to reviewers comments_v5.docx [file pgph.0005246.s005.docx]

**PGPH-D-25-00886R2	August 2025**

**Is household contact investigation a missing link for tuberculosis care in Chhattisgarh, India? – Operational research using programmatic data**

PLOS Global Public Health

**Sonali Sarkar
Academic Editor
PLOS Global Public Health**

**Subject**- Point-point response to the reviewer’s comments

Thank you for the email communication on 19^th^ August 2025 and for providing comments and suggestions. We have carefully read all the comments and made relevant changes in the manuscript. We have attempted to respond to each of the points raised. The editor’s comments are copied below, and the responses provided point by point.

We hope that the responses in the letter and changes made in the revised manuscript are to the satisfaction of the reviewer and editor. We appreciate the effort put in by both of you in providing us with a valuable peer review to improve the manuscript.

Yours sincerely,

Chetanya Malik

Corresponding author

Journal Requirements:

If the reviewer comments include a recommendation to cite specific previously published works, please review and evaluate these publications to determine whether they are relevant and should be cited. There is no requirement to cite these works unless the editor has indicated otherwise. 

Please review your reference list to ensure that it is complete and correct. If you have cited papers that have been retracted, please include the rationale for doing so in the manuscript text, or remove these references and replace them with relevant current references. Any changes to the reference list should be mentioned in the rebuttal letter that accompanies your revised manuscript. If you need to cite a retracted article, indicate the article’s retracted status in the References list and also include a citation and full reference for the retraction notice.

**Author’s comment- The reference list has been rechecked as per the editor’s suggestion in the revised** **manuscript.**

**Additional editor’s comment-**

Thank you for modifying the manuscript as per the suggestions of the reviewers. However, a thorough language check will improve the quality of the manuscript. Please modify the following portions.
1. Lines 230-233, where an explanation has been provided for the discrepancies in the numbers of the HHCs investigated. There is repetition of information in the two sentences.

**Author’s comment- Lines 230-233 have been modified, and the repetition of information has been removed for better clarity in the revised manuscript with track changes lines 247-253.**

2. Lines 261-266 need to be simplified to convey the message clearly. You may consider the following to replace these lines.
"Lack of communication between the tertiary care centres and private health care providers with the functionaries at PHC responsible for HHC investigation, inadequate training for primary health care workers, poor data quality, or a combination of the above may have contributed to HHC investigations not being done."
**Author’s comment- Lines 261-266 have been modified as per the editor’s suggestion in lines 279-286 of the revised manuscript with track changes.**
Overall, the number of words and sentences can be reduced to make the manuscript succinct.

**Author’s comment- We have tried to make it more succinct as per the suggestion**.
